# Continuity of clinical management and information across care levels: perceptions of users of different healthcare areas in the Catalan national health system

**DOI:** 10.1186/s12913-016-1696-8

**Published:** 2016-09-02

**Authors:** Sina Waibel, Ingrid Vargas, Marta-Beatriz Aller, Jordi Coderch, Joan Farré, M. Luisa Vázquez

**Affiliations:** 1 Health Policy and Health Services Research Group, Health Policy Research Unit, Consortium for Health Care and Social Services of Catalonia, Av. Tibidabo 21, 08022 Barcelona, Spain; 2 Department for Paediatrics, Obstetrics and Gynaecology, Preventive Medicine, Universitat Autònoma de Barcelona, Av. de Can Domènech 737, Bellaterra (Cerdanyola del Vallès), 08193 Spain; 3 Grup de Recerca en Serveis Sanitaris i Resultats en Salut, Serveis de Salut Integrats Baix Empordà, Carrer Hospital 17-19 Edifici Fleming, Palamós, 17230 Spain; 4 Centre Integral de Salut Cotxers, Av. de Borbó 18 - 30, Barcelona, 08016 Spain

**Keywords:** Continuity of patient care, Qualitative research, Quality of health care, Health information management, Patient care management, Patient navigation

## Abstract

**Background:**

The integration of health care has become a priority in most health systems, as patients increasingly receive care from several professionals in various different settings and institutions, particularly those with chronic conditions and multi-morbidities. Continuity of care is defined as one patient experiencing care over time as connected and coherent with his or her health needs and personal circumstances. The objective is to analyse perceptions of continuity of clinical management and information across care levels and the factors influencing it, from the viewpoint of users of the Catalan national health system.

**Methods:**

A descriptive-interpretative qualitative study was conducted using a phenomenological approach. A two-stage theoretical sample was selected: (i) the study contexts: healthcare areas in Catalonia with different services management models; (ii) users ≥ 18 years of age who were attended to at both care levels for the same health problem. Data were collected by means of individual semi-structured interviews with patients (*n* = 49). All interviews were recorded and transcribed. A thematic content analysis was conducted segmented by study area, with a mixed generation of categories and triangulation of analysts.

**Results:**

Patients in all three areas generally perceived that continuity of clinical management across levels existed, on referring to consistent care (same diagnosis and treatment by doctors of both care levels, no incompatibilities of prescribed medications, referrals across levels when needed) and accessibility across levels (timeliness of appointments). In terms of continuity of information, patients in most areas mentioned the existence of information sharing via computer and its adequate usage. Only a few discontinuity elements were reported such as long waiting times for specific tests performed in secondary care or insufficient use of electronic medical records by locum doctors. Different factors influencing continuity were identified by patients, relating to the health system itself (clear distribution of roles between primary and secondary care), health services organizations (care coordination mechanisms, co-location, insufficient resources) and physicians (willingness to collaborate, commitment to patient care, the primary care physician’s technical competence).

**Conclusions:**

Care continuity across care levels is experienced by patients in the areas studied, with certain exceptions that highlight where there is room for improvement. Influencing factors offer valuable insights on where to direct coordination efforts.

**Electronic supplementary material:**

The online version of this article (doi:10.1186/s12913-016-1696-8) contains supplementary material, which is available to authorized users.

## Background

The lack of integration of health services is considered to be one of the key causes of poor quality of care [1, 2]. Fragmented care, or care that is insufficiently coordinated between providers, might be harmful to patients due to the duplication of diagnostic tests, inappropriate poly-pharmacy and conflicting care plans [1]. Especially patients suffering from multi-morbidities and chronic conditions tend to receive care from several professionals of different disciplines in various settings and institutions, thus meeting their healthcare needs through seamless care over extended periods of time is particularly challenging [3, 4].

The analysis of integrated care should take into account intermediate outcomes (access to healthcare, care coordination and continuity of care) and final outcomes (equity of access, efficiency and quality of care), as well as the factors influencing it, from different perspectives, including those of the key actors [5]. Analysing continuity of care from the patient’s perspective endows one main benefit: they can provide a global picture of the care provided since they experience services along the continuum of care [6] and first-hand from multiple providers [7]; hence seeking patients’ perceptions and experiences provides an excellent opportunity to better understand this phenomenon [8, 9].

The concept of continuity of care has been garnering more attention in recent years in line with the publication of several meta-syntheses that aim to improve the conceptual framework [10-14] and clarify the conceptual boundaries of related terms, such as care coordination or integration [15]. Although conceptual discussions are ongoing, an increasing number of qualitative and quantitative studies, for example those of Cowie et al. [16], Aller et al. [17, 18] or Uijen et al. [19], have adopted Reid et al.’s framework [4, 9]. They define continuity of care as one patient experiencing care over time as connected and coherent with his or her health needs and personal circumstances [20]. In other words, continuity of care can be understood as the result of care coordination as experienced by an individual patient [20]. Their model classifies continuity according to three types [4, 9]: *continuity of clinical management*, which is the patient’s perception that they receive the different services in a coherent way that is responsive to their changing needs; *continuity of information*, which is the patient’s perception that information on past events and personal circumstances is shared and used by the different providers; and *relational continuity*, which is the patient’s perception of an ongoing therapeutic relationship of the patient with one or more providers. Continuity of clinical management and continuity of information can be analysed across levels of care (primary and secondary care interface) and will thus be the focus of this article.

Recent literature reviews [6, 12] confirm that a number of qualitative studies have analysed patients’ experiences and perceptions of continuity of care. These studies, mainly conducted in the NHS of the United Kingdom and Canada [6, 12], concentrated on relational continuity, whereas continuity of clinical management and information were studied only in a limited way [12]. Furthermore, most studies focused on chronic conditions, especially diabetes [6, 12], cancer [6], and mental health problems [6, 12]. Therefore, perceptions of patients with acute health problems or different conditions receiving care from both care levels have been analysed to a lesser extend, which would allow for identifying context-specific shortcomings concerning smooth transitions.

The analysis of the association between the perception of continuity of care and factors which could influence (i.e., facilitate or hinder) continuity of clinical management and continuity of information from the patients’ perspective focused on individual factors using quantitative methods. Results suggested differences related to age and educational level – the elderly population is more likely to perceive higher levels of continuity of clinical management and information [7, 18, 21], whilst higher education was significantly associated with lower ratings [21] – but the influence of socioeconomic level, health status or sex on the continuity types is inconclusive [7, 18, 21]. The analysis of factors related to health services organizations and health professionals was targeted in two quantitative studies; one conducted in Catalonia, Spain [17] and the other in Quebec, Canada [22]. Results showed that healthcare areas (which differed in the management model of primary and secondary care) [17] and operational agreements with other healthcare establishments (mostly shared-care protocols and mechanisms for facilitated referrals and information sharing) were associated with all three continuity types [17, 22]. In qualitative research, influencing factors predominantly emerged as a by-product of the study results, i.e., their identification was not the study objective, with a few exceptions [11, 13, 23, 24], or seemed to be entangled with the elements that defined continuity. Moreover, it is not clear to what extent the factors were actually mentioned by patients or whether they were inferred in the interpretation of the researchers. 

Scant evidence exists on the possible consequences or outcomes of (dis)continuity of care [11]. According to a meta-analysis, the association of continuity of clinical management and information with reduced health services utilization (including hospitalization and emergency visits) and patient satisfaction is uncertain [25]. Qualitative research suggests that coordination breakdowns lead in particular to the transfer of responsibilities to patients, for example, the patients having to act as a mechanism of coordination to maintain the continuity of clinical information [24] (“patients as the information broker” [23]) and keep all agencies abreast of changes [26]. From the professionals’ point of view, this could produce significant medical errors [27]. Thus, although continuity of care is already purported to be a critical feature of high quality services, more documentation is needed to better explain the relationship between continuity and its potential outcomes [28].

Achieving integrated care has also become a priority of the Spanish NHS, which is financed by taxes and decentralized into regional health services. At the time of the study, it offered universal coverage and free access at point of delivery [29]. Healthcare provision is organized into levels of complexity: primary care is the gatekeeper and is responsible for coordinating the patient’s care along the care continuum [30] and secondary or specialist care acts as a consultant for primary care and is responsible for more complex care [29, 31]. In the autonomous community of Catalonia, the healthcare system is characterised by a split of the financing and provision functions. The provision of services is the responsibility of a number of contracted providers; mainly the Catalan Health Institute (*Institut Català de la Salut*), but also consortia, municipal foundations and private foundations (largely non-profit but also for profit), which make up the Integrated Healthcare System for Public Use (*Sistema sanitari integral d’utilització pública de Catalunya*) [32]. This diversity of providers has originated various management models for the joint management of primary and secondary care, such as integrated healthcare networks [33]. In Catalonia, empirical evidence on the three continuity types is scant, except for a population survey using the CCAENA© questionnaire [17, 18] and a qualitative multiple case study with COPD patients attended to at different integrated healthcare networks [34]. Other surveys of the Catalan NHS – the CatSalut Satisfaction Survey Plan (PLAENSA©) [35] and the Health Survey of Barcelona [36] – seek the citizens’ perception in terms of quality and satisfaction and thus include only specific items related to continuity of care, for instance information transfer across levels or waiting times for a secondary care visit. Results suggest that transitions between primary and secondary care were mostly reported by patients to be connected and consistent; however some noteworthy elements of discontinuity were identified, for example long waiting times for secondary care after a referral [17, 34] or gaps in the information transfer across levels [17]. The need for an in-depth analysis to explore the rationales that could explain these results and thus to understand the full complexity of the phenomenon has been postulated [17].

The aim of this study is to analyse perceptions of continuity of clinical management and information across care levels and the factors influencing it, from the viewpoint of users of the Catalan NHS. This article forms part of a wider study that analyses the relationship between coordination and continuity across care levels by seeking the health care services’ [37] and users’ perspectives (here presented).

## Methods

### Study design

A descriptive-interpretative qualitative study was conducted with healthcare users using a phenomenological approach. Studies that draw upon the phenomenological perspective concentrate on exploring how individuals make sense of the world in terms of the meanings and classifications they employ [38]. To orient the study of the phenomenon – continuity of care – the conceptual framework created by Reid et al. [4] was employed.

### Study sample

A two-stage theoretical sample was designed. In the first stage, the study contexts were selected to represent the diversity of management models in the Catalan NHS. Three healthcare areas were chosen: the Baix Empordà region, the city of Girona and Ciutat Vella in Barcelona (Table 1). In Baix Empordà and Girona, primary and secondary care services are managed by the same entity; under private law in the former case and under public law in the latter. In Ciutat Vella, two entities manage primary care and a different entity manages secondary care; under private and public law. All three areas have implemented similar mechanisms for clinical coordination across levels, such as shared clinical guidelines and protocols, virtual curbside consultations of primary care doctors with specialists, periodic discussion of clinical cases and automatic notification of hospital discharge for primary care follow-up. The information system implemented, however, differs according to the area (i.e., one/two shared/not shared electronic medical record system(s)).

**Table 1:** Description of study areas

	Baix Empordà region	City of Girona	Ciutat Vella of Barcelona
Population^a^	74,144	83,312	99,093
Location	Rural and semi-urban	Urban	Urban
Primary care providers			
Number of basic health zones	4	4	5
Managing entity/entities	SSIBE	ICS	ICS (4 zones) PAMEM (1 zone)
Secondary care providers			
Number of hospitals	1	1	1
Managing entity	SSIBE	ICS	PSMAR
Information system	Single shared EMR system	Two shared EMR systems	Two EMR systems; not shared (ICS with PSMAR) and shared (PAMEM with PSMAR)

^a^Population ≥ 18 years of age; Source: Registro Central de Asegurados 2010 [62]

*EMR* electronic medical records, *ICS* Institut Català de la Salut, *PAMEM* Institut de Prestacions d’Assistència Mèdica al Personal Municipal, *PSMAR* Parc de Salut Mar, *SSIBE* Serveis de Salut Integrats Baix Empordà

In the second stage, in each context, the informants were selected according to the following criteria: healthcare user of 18 years of age or over who had been attended to in both primary and secondary care for the same health problem within the three months prior to the interview. Secondary care included specialist (outpatient and inpatient) and emergency care. Variation criteria were considered during the selection process (taking into account sex, age, country of origin and the use of different services) in order to take in a broad set of data and experiences (maximum-variation sampling [39]). The participating organizations provided a list with basic demographic data of users who met the established criteria to the first author, who then selected the informants taking variation criteria into account. One respondent was recruited through a snowballing technique [39], i.e., asking a study participant to identify others who met the defined criteria and whose experience would be relevant to the study.

The final sample consisted of 49 users, between 14 and 18 per area (Table 2). Slightly more than half were female. The age of the participants ranged from 22 to 82 years of age. Thirteen users were foreign-born, mainly originating from Latin America (*n* = 7), but also some from North Africa (*n* = 3), other European countries (*n* = 2) and Asia (*n* = 1). The sample represented users of all socio-economic statuses (from unskilled to non-manual and high-level professionals, as well as short and long term unemployed, retirees and housewives). Most of the informants who were unemployed were also foreign-born (*n* = 7).

**Table 2:** Characteristics of the sample

	Baix Empordà region	City of Girona	Ciutat Vella in Barcelona
Female	9	8	10
Age	33–82	22–82	26–70
Foreign-born	4	4	5
Unemployed	1	4	5
Total	18	14	17

### Data collection

The data were collected through individual semi-structured interviews. An interview topic guide on continuity of care was drawn up containing two main parts: a general part about the user’s health status (serving as an icebreaker) and their experiences with the healthcare services, and a specific part about their perceptions of continuity of care (please see Additional file 1 - Interview guide). The latter part was oriented by Reid et al.’s [4] conceptual framework and included questions to explore the different dimensions of perceived continuity of clinical management across levels (opinions on consistency in diagnosis and treatment, collaboration between physicians, referrals and accessibility) and continuity of information across levels (information transfer and use). The topic guide evolved throughout the data collection process according to the initial analysis and was used as a prompt for the researchers to ensure that relevant topics of continuity of care were covered. New themes were also pursued as they came up over the course of the interviews.

Selected users were sent an initial invitation letter and then contacted by telephone either by staff of the healthcare organization or the first author. Interviews were conducted in the patients' preferred location, in most cases at their home but also at their workplace, in a café or a quiet room at the primary care centre. Interviews ranged from 35 to 75 min long and lasted one hour on average. In some cases, the user’s carers provided relevant opinions, which were included in the analysis. All interviews were audio taped, transcribed in full, anonymized, and checked against the tape by the first author. Data collection stopped when saturation was reached in each area, which was when encounters with new participants no longer elicited themes that had not been raised by previous participants [39]. Field notes on preliminary ideas and reflections were made continuously to enhance reflexivity, thus acknowledging the influence a researcher has on the research process [39]. Fieldwork was conducted from March 2011 to March 2012.

### Data analysis

A thematic content analysis was conducted by the first author using the software Atlas-ti 5.0. Data were segmented by study area. Following initial familiarization with the interview contents, a mixed generation of categories took place, i.e., we based our analysis on the categories used in the topic guides but left room for new categories to emerge [40]. Transcripts were coded and categories were developed and refined as new sections of text were examined. The final list of categories consisted of the perception of the existence of each continuity of care dimension across levels and its defining elements, influencing factors and consequences. Results were triangulated by three researchers who were knowledgeable about qualitative research and the phenomenon of the study.

## Results

### Perception of the existence of care continuity across levels of care

Patients in all three healthcare areas generally perceived that *continuity of clinical management across care levels* existed because they had received *consistent care* from the primary care physician (PCP) and the secondary care physician (i.e., same diagnosis and treatment from doctors of both care levels without conflicts; no unnecessary repetition of tests; referrals across care levels when needed). Furthermore, they considered that *care was accessible* (i.e., waiting times after a referral in accordance with their health needs). Only a few patients across all areas identified elements of discontinuity in clinical management, such as differences of opinion on diagnosis, missing or delayed referrals to secondary care or excessive waiting times to have specific tests performed. With regard to *continuity of information across care levels*, patients confirmed that *information was transferred and used* via computer by their regular doctors, with the exception of patients assigned to one of the two selected primary care providers in Barcelona. A few patients in all areas highlighted a limited uptake of information by some locum and emergency doctors. (Please see Additional file 2 for subcategories and additional quotations and Additional file 3 for the original Spanish language version of the quotations).

#### Receipt of consistent treatment across levels with some contradictions, duplications and missing referrals

Patients across all the healthcare areas largely perceived that the care provided by their primary and secondary care physician was consistent because they received the same diagnosis, treatment and medical recommendations and did not experience any incompatibilities of prescribed medications: *They check to see if one medicine is compatible with another. Because I take (acenocumarol) and that also makes medication much more complicated. A lot of medicines aren’t compatible with (acenocumarol) and they have to keep a constant eye on all that (Barcelona, male patient, 70).* A few patients across the areas, however, experienced inconsistencies, reporting that they had received different opinions on the diagnosis and the severity of their condition. Receiving different opinions left patients feeling ‘alone’ or ‘in limbo’. Furthermore, a few patients reported that the emergency doctor prescribed drugs which were different from those recommended by the PCP and resulted in secondary effects. Finally, in a few cases, physicians failed to communicate with each other to adapt the treatment plan or solve the health problem together.

Patients across the organizations considered that tests for diagnosis or follow-up were only repeated in the other care level when necessary, i.e., when the results were insufficient to reach a diagnosis or the diagnosis needed to be confirmed by the specialist: *I go to emergencies (in the primary care centre), right? And I have some tests done (…) and then if I go over there to Palamós (hospital), they do the same tests again. Over there they want to see these tests because of course they have to check that this is the case (…) Well, of course the specialist has to be completely sure of what needs to be done (Baix Empordà, male patient, 75).* In a few cases only, but in all the areas, patients highlighted that tests should not have been repeated, citing expired pre-operatory results or excessive processing times in the delivery of diagnostic results for acute symptoms to primary care, resulting in the patient having to repeat them in the emergency department.

With regard to adequate transitions across levels, patients generally highlighted that they were sent to the right speciality of secondary outpatient care and were referred whenever it was necessary, i.e., when a diagnosis needed to be made or confirmed, a specialist test done or the medication plan modified. Patients were referred on the basis of the PCP’s or the patient’s identified need: *To tell the truth, whenever I feel that my GP doesn’t really know for sure what to do or where to go from here, and I ask him to refer me to somewhere in particular, the truth is he agrees to it (Girona, male patient, 47)*. Several patients across the areas, however, felt that the PCPs had put off their referral or failed to refer them altogether, either due to excessive waiting times for secondary care or because they were trying to solve the health problem by themselves. Perceived delayed or absent referrals resulted in some patients seeking private care. Moreover, it was seen to be a waste of time and resources as it made additional primary care visits necessary. On the topic of primary care follow-up after a secondary care visit (outpatient, inpatient or emergency care), patients of all areas considered that they were referred to primary care when necessary, i.e., to have clinical tests performed, test results entered into the EMR system (in Barcelona), to be afterwards sent to secondary outpatient care following a visit to A&E and to have the medication plan adapted (reconciled) or new medication prescribed: *From emergencies (…) they send you home and they say “right, go and see your GP tomorrow”, because of course normally it’s your GP who has to see you and prescribe the medicine you need. It’s worked really well like that for years now (Girona, female patient, 66)*. Furthermore, patients considered that primary care follow-up was adequate since they themselves arranged a visit when required (own initiative), which was usually to inform the PCP of the preceding secondary care visit.

#### Timely access across levels with the exception of waiting times for specific tests

Patients across all three areas generally agreed on the adequate timeliness of *non-urgent* secondary outpatient care after a referral from the PCP. Waiting times depended on the speciality and ranged from a few days (to see the ear, nose and throat specialist or gynaecologist) to a couple of weeks or a month (to visit the pulmonologist, ophthalmologist, dermatologist or internal specialist), which however was considered to be short or reasonable due to its non-urgent nature: *It was a reasonable time to wait (…) maybe a month or so, more or less, but as it wasn’t anything important I think the waiting time was OK (Baix Empordà, female patient, 46).* Furthermore, waiting times were perceived to be adequate due to the possibility of seeking emergency care if needed. Similarly, patients highlighted that they were seen quickly for *urgent* primary care referrals to secondary outpatient or emergency care (perceived to be required when they were in a life-threatening situation, suffered pain and had to take pain killers, or the condition prevented them from working). Some patients across the areas, however, pointed out that waiting times for specialist care were too long, especially for certain specific tests (for example, one month for magnetic resonance imaging or ultrasound scans and three to six months for X-rays and polysomnograms), as well as for surgery (e.g., breast reconstruction or cataract surgery); the latter being a major concern of patients in Girona who pointed out that slow access could cause their condition to deteriorate. Patients perceived long waiting times to be worse when they lacked information on the possible date of surgery and medical recommendations on how to best get through the waiting times. As a consequence of long waiting times, patients either reported interruptions in the diagnostic process and treatment or indicated that the PCP tried to counteract this situation by taking on the specialist’s tasks (such as diagnosing or performing tests), making urgent referrals even for minor health problems or recommending to the patient to seek emergency care. Furthermore, patients expressed fears of losing their job when on sick leave for a longer period and pointed out that not knowing the diagnosis caused anxiety: *They told me (in primary care) that the MRI would be done by the end of January (…) about a month away, but imagine a month with my leg like that, in loads of pain and not knowing what was wrong or anything, you know? And it was… worrying (Barcelona, female patient, 37).* Subsequently, some patients sought private health care either in Catalonia or, in the case of some foreign nationals, in their country of origin.

With reference to waiting times for primary care after a specialist consultation or performed complementary tests, patients highlighted that they were seen rapidly, given that they themselves could program the visit with their PCP on the date needed: *Going back to the GP, well, you just get an appointment for the next day, don’t you? When you’re told “you’ll have the ultrasound in four weeks’ time” then you know that one or two days after you get an appointment with your GP (Barcelona, male patient, 54).*

#### Sharing of information across levels of care principally via electronic medical records

Patients largely perceived that clinical information was registered in their medical records, transferred via computer and accessible to physicians of both care levels, since there was no need to comment on antecedents or a preceding visit, nor to pick up, store and deliver paper-based test results, medication plans or discharge reports to the physician of the other care level. Patients from Girona in particular observed positive progress in information sharing in the last few years: *Now on an information level, it’s all been digitalized pretty well, and the GP also has access to the tests I have done at the hospital (…) where before you had to go around with a (fat) file like this with X-rays in it (Girona, male patient, 79)*. An exception to this emerged in the discourse of patients served by one of the two primary care providers studied in Barcelona, where most patients reported that the computers were not ‘interconnected’ across care levels, thus information was not shared automatically. Nevertheless, a number of patients from this area pointed out that test results, discharge reports and medication plans were transferred from secondary to primary care since patients themselves carried that information to the other care level.

Patients across all three areas confirmed that doctors consulted the information registered in their clinical records before or during the consultation and thus were aware of their antecedents or the motive of the visit. A few patients mentioned that information availability and uptake avoided the duplication of tests and the prescription of incompatible drugs: *Of course they’ve got it (the information in A&E) because if they didn’t it would be dangerous for the patient. Suppose they inject you with some medicine that reacts with what you’re already taking. No no, it’s sacred (Girona, female patient, 66)*. Moreover, information transfer via computer speeded up the visit since there was no need to explain health problems again and reduced the need for informal communication (via e-mail or telephone).

Nonetheless, a very small number of patients across the three areas highlighted that specific information was not shared via computer in certain settings, e.g., the previous clinical events and secondary care discharge reports were not shared with primary care, or medication plans with emergency care. Furthermore, emergency and locum doctors of both care levels did not always consult the information stored in their records: *There was a substitute doctor (…) and she clearly couldn’t be bothered with all those medical records. I came to see her because… I think I had to explain it to her (Girona, female patient, 55)*. Inexistent or limited information transfer resulted in avoidable referrals, the unnecessary repetition of tests, and the prescription of incompatible treatments.

### Perceived factors influencing care continuity across levels of care

**Fig. 1:**
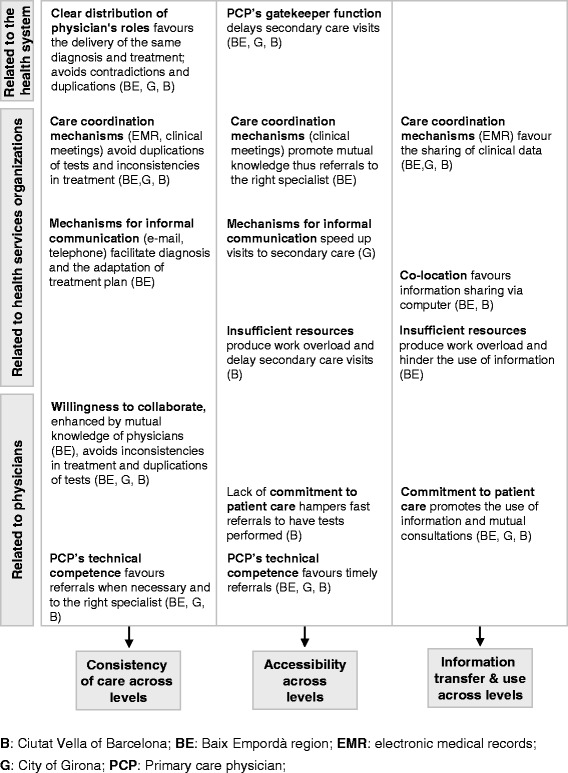
Factors influencing continuity of care across levels in the study areas

The existence of continuity of care was linked by patients across all the study areas to a variety of influencing factors, which were related to the health system, health services organizations and physicians. The factors related to the health system and physicians emerged in all areas; whereas certain factors related to health services organizations were specific to the area in which the patient was served. (Please see Fig. 1, Additional file 2 for subcategories and additional quotations and Additional file 3 for the original Spanish language version of the quotations).

#### Influencing factors related to the health system

Patients across the study areas considered that the *clear distribution of roles* and responsibilities between primary and secondary care favoured consistency in diagnosis and treatment, and avoided incompatibilities of prescribed medication and the duplication of tests. The secondary care physician was perceived to be responsible for recommending drugs and modifying the treatment and the PCP for updating the medication plan according to the specialist’s instructions, and for handing over the prescription: *Nobody changes the medication except for the nephrologist and the internal medicine doctor (…) the GP is the one who has to make out the prescriptions for chronic illnesses, he doesn’t make any adjustments to the medication. The GP just coordinates it, so to speak. (Baix Empordà, female patient, 43)*. Furthermore, roles were clearly distributed for performing tests and following-up the patient’s condition, with either the primary or secondary care doctor being in charge. Whilst most patients believed that the *PCP’s gatekeeper function* guaranteed adequate access to secondary care (being referred only when necessary and to the right speciality), some patients considered that the PCP insisted on solving the health problem by him-/herself and thus did not refer the patient initially, and others highlighted that primary care visits were an unnecessary intermediate step that only served to increase waiting times for secondary care: *I think it would be better if you could go straight to the specialist because it saves a lot of time (…) if it’s already clear to you that you need to go to a specialist, it’s silly that you should have to wait for the doctor to say “ah yes, well you do have to go” (Baix Empordà, female patient, 43).*

#### Influencing factors related to health services organization

*Care coordination mechanisms*, particularly EMR implemented in most areas, facilitated information sharing across levels, thus avoiding the unnecessary duplication of tests and inconsistencies in treatment: *When I go to cardiology, he opens my record and sees all the medication that I take (…) My GP, she knows it all too, she presses a button and it’s all there in the records. And they also check to see that one medicine is compatible with another (Barcelona, male patient, 70).* Face-to-face meetings (clinical meetings) of physicians of the two care levels, a care coordination mechanism mentioned by patients attended to in Baix Empordà, was believed to lead to mutual knowledge of physicians, which facilitated referrals to the right specialist and the adequate and mutual adaptation of the medication plan. Some patients, again particularly those of Baix Empordà, highlighted that *mechanisms for informal communication* (use of e-mail and telephone) were used for curbside consultations, which speeded up and facilitated diagnosis and the mutual adaptation of treatment. Moreover, the use of informal communication for making patient referrals shortened waiting times for secondary care, as highlighted by patients from Girona.

A few patients indicated that the *co-location* of secondary care physicians in primary care centres in Barcelona and in Baix Empordà (physiotherapists only in the latter case) favoured information sharing since the patient’s EMR were accessible to all doctors working in the same facility: *I suppose so (there is communication) because I know that the physiotherapists go to the primary care centre because when I had a problem with my back, I asked the physio: “Do I need to take the report up to my GP” and she said “no, because we communicate with each other”, and anyway I suppose if she opens her computer she sees it too (Baix Empordà, female patient, 42).*

*Insufficient resources,* demonstrated by a shortage of doctors and translating into work overload, was considered to be an important cause of long waiting times for secondary care in Barcelona and was also related to the insufficient uptake of information from the clinical history by locum doctors in Baix Empordà. A few patients in Barcelona reported that the budget cuts during the recent economic crisis led to insufficient resources and a consequent reduction in staff: *If it (the health system) isn’t working well at the moment it’s because of the (budget) cuts and because the health workers and doctors are overloaded (…) so then we all suffer around here, both health professionals and patients (Barcelona, female patient, 52).*

#### Influencing factors related to physicians

Patients across the areas considered that the physician’s willingness to collaborate with colleagues, by providing test results for the other care level and using them, avoided duplication of tests. Furthermore, taking into account instructions and recommendations from physicians of the other care level avoided inconsistencies in treatment: *(The neurologist) told me to either reduce the dosage of this medicine or change it for another one, but always keeping in mind what my GP said (…) So the neurologist, on checking the medicine that my doctor had recommended, he said: “OK, well let’s take this one away as it’s not working anymore.” (Barcelona, male patient, 48).* This willingness to collaborate was reinforced by physicians knowing each other (mutual knowledge) and meeting up to discuss cases, as was mentioned by patients from Baix Empordà.

The physician’s *commitment to patient care* and interest in solving the health problem was linked by patients across the three areas to the uptake of information from EMR and consultations with other physicians to solve the health problem together, which was facilitated by mutual knowledge: *I’ve been in Barcelona for 30 years and yes (there is communication), more or less, but everyone knows each other around here. The doctors consult each other a lot, because they care about the patient’s wellbeing, so that’s the priority around here, that the patient is OK. (Baix Empordà, female patient, 33).* A lack of commitment on the part of the PCP was associated with delays in perceived necessary referrals to secondary care.

Lastly, patients across the study organizations generally felt that PCPs had the *technical competence* to evaluate whether a referral was necessary and to send the patient to the right speciality, thus resulting in adequate referrals to secondary care: *The GP, he’s the one who’s got the knowledge at his fingertips. First you see your GP; he knows who needs to be called and how to get an appointment (…) just fine (Baix Empordà, male patient, 81)*. Furthermore, PCPs were well informed on the urgency of the referral, which had a positive effect on accessibility to secondary care.

## Discussion

Few studies have analysed continuity of care across the primary and secondary care levels from the perspective of patients with different health conditions, and even fewer have focused on the factors influencing the different types of continuity. This study contributes to filling the existing knowledge gap by exploring continuity through a qualitative study with healthcare users in three areas of the Catalan NHS with different services management models.

### Perceived continuity of care across levels with a few interruptions

Patients across the three study areas generally perceived consistent care across levels, results which were largely in keeping with those of a survey of users conducted in the same study areas [17, 18] and a qualitative multiple case study of COPD patients served in Catalan integrated healthcare networks [34]. Patients reported that they had received the same diagnosis and treatment from physicians of the different care levels, without inconsistencies or unnecessary repetitions of tests, and with referrals to the other care level when necessary. Nevertheless, a few patients in all areas identified specific elements of discontinuity, such as long waiting times for specific tests or the insufficient use of EMR by locum doctors. Furthermore, according to the accounts of a few patients, they themselves made coordination between services happen by delivering discharge reports and medication plans across care levels, which point to additional coordination breakdowns, even though the patients did not quoted negatively about continuity of information. A number of qualitative studies have described difficulties in delivering continuity of clinical management, e.g., missing referrals between different centres in Australia [41] or services following discharge not being arranged in the United Kingdom [42]. These failures could also be explained by the argument that patients notice poor continuity in particular [6, 43–45], or that studies were specifically aimed at identifying breakdowns in order to improve health services, e.g., by means of better cancer care coordination [41].

With regard to access across levels, patients generally highlighted the adequate timeliness of appointments after a referral (except for certain specific tests). In the abovementioned Catalan user survey, nearly half of the interviewees (42 %) held the opinion that waiting times for secondary care after a referral were long or excessive [17]; and a meta-synthesis of the relevance of continuity of care in cancer treatment highlighted the need to shorten waiting times for secondary care [46]. Our result of a general positive perception does not necessarily contradict previous studies if we take into consideration the additional information provided by patients on the perceived urgency of the visit: extensive waiting times for secondary care were thought to be acceptable for minor health problems by virtue of their non-urgent nature and the possibility of seeking emergency care if the condition worsened. Furthermore, patients were more likely to accept long waiting times when provided with useful information (estimated waiting times, as stated elsewhere [47], and treatment recommendations to get through the waiting times). For urgent referrals, waiting times were considered to be short, as perceived by patients with COPD, who highlighted rapid or immediate access to primary and secondary care during exacerbations [34].

With reference to continuity of information, patients across the areas perceived the availability of clinical information across levels (mainly via computer) and its adequate uptake; a result in keeping with those of the case study of COPD patients in Catalonia [34]. In contrast, numerous international studies, for example those conducted in England [16, 48] or the United States [7, 49], suggest significant communication breakdowns as observed by patients [7, 16, 48] and providers [49], thus results are highly context dependant. One exception to the fact that information transfer was generally perceived to exist emerged from the discourse of users assigned to Ciutat Vella in Barcelona, where information was perceived to be only partially shared across levels. This finding was also confirmed in the population survey [18]. Given that Ciutat Vella differs from the other study areas in terms of the information system implemented, we may infer from this that patients are able to notice the care coordination mechanisms put in place [8, 50].

### Factors favouring or hindering continuity of care across levels

The factor that emerged with greatest intensity in the patients’ discourse was one related to the ***health system***: the *clear distribution of roles* between primary and secondary care physicians, which was considered to favour all elements of consistency of care and access across levels. The concept of ‘role clarity’ emerged as a recurrent topic in a meta-synthesis [6], but only with reference to discontinuity: the provision of conflicting information was linked to physicians’ unclear roles, which shook the patients’ faith in the doctors’ overall competence and expertise [6]. Given that patients in all three study areas, in addition to COPD patients served in integrated healthcare networks [34], considered that role clarity favoured continuity, the results appear to indicate the adequacy of the healthcare model based on primary health care as promoted by the Spanish and other NHS, by enhancing the delivery of care at the right care level and the coordination of care along the care continuum with primary care exercising the *gatekeeper and coordinator functions*. According to two cross-sectional studies conducted in the United States, patients reported higher continuity of clinical management and information when the specialist visit was based on a PCP referral [7]; and almost all patients valued the PCP's role as a source of first-contact care and coordinator of referrals since they were not always aware of when a referral was clinically indicated or appropriate, whereas the PCP was perceived to be qualified to manage their care across levels [51]. Similarly, in our study, patients generally highlighted that adequate and timely referrals were attributed to the PCP’s technical competence. Nevertheless, as it emerged in the discourse of some patients, the gatekeeper system could also extend waiting times or even fail to provide access to secondary care when the PCP insisted on solving the health problem by him-/herself; hence the gatekeeper system might also act as a barrier to accessing secondary care. In the United States, twelve percent of patients belonging to managed care plans reported that it was difficult to get the specialist referrals they needed, leading to lower ratings of trust, confidence and satisfaction with the PCP [51]. Particularly Spanish individuals belonging to lower socioeconomic groups might be affected by the PCP’s unfavourable referral pattern [52] and should hence be paid special attention so that their health needs are met.

***Health services organizational*** factors (care coordination mechanisms, co-location and insufficient resources) were largely identified to be specific to one or two study area(s), particularly to Baix Empordà, except for the implementation of an EMR system, which was mentioned across all three areas and which, if used adequately, might be the key mechanism to enhance continuity as perceived by patients. *Co-location*, i.e., specialists located in the primary care centres, emerged to be an important mechanism in Barcelona that favours information sharing given that physicians had access to the same clinical history. Co-location of providers was a factor mentioned previously in qualitative studies conducted in Canada on continuity of care, however, in the context of enhanced interactions and coordination between physicians [23, 24] and accessibility to services [23]. These results are similar to those of a study on care coordination (perspective of physicians) conducted within the framework of the same reserach project, suggesting that physical proximity between physicians increases mutual knowledge, which in turn fosters a more favourable attitude towards coordination. With reference to the identified factor *insufficient available resources*, a few patients considered that the reduction of the healthcare budget in Spain in 2012 as a result of the economic crisis [53] resulted in work overload and lack of time and hindered the uptake of information from clinical histories, especially by locum doctors, and the use of curbside consultations, as it has also been shown in a Belgium study [54]. Nevertheless, the impact of the budget cuts on continuity of care emerged with low intensity in the patient discourse; presumably because data was collected at the onset of the crisis. It is therefore logical to assume that the crisis would have appeared more relevant if interviews had been conducted at a later point in time.

Patients identified three factors related to ***physicians*** that influence continuity of care (*willingness to collaborate*, *commitment to patient care*, and *the PCP’s technical competence*). In spite of being less salient in the patients’ discourse than factors related to the health system and health services organizations, they are most likely just as relevant for achieving continuity. To the best of our knowledge, literature on this topic, when concerning the patient’s perspective, is rare; except for the motivation to work cooperatively [13]. The factor ‘values and attitudes with regard to coordination’ also emerged in the discourse of physicians (explored in the same study areas) and was perceived to influence the use of the different coordination mechanisms implemented.

### What are the consequences of continuity of care?

Even though only a few *dis*continuity elements were identified by patients, important consequences resulted from those and should thus be paid attention. Lack of consistency of care and access across levels was linked to the inadequate use of resources, seeking private care (due to absent referrals or long waiting times), feelings of loneliness and anxiety (due to receiving different opinions and long waiting times, respectively) and most importantly, as mentioned by only a few informants, potential negative health effects (adverse secondary effects when receiving different prescriptions or deterioration of condition when access to secondary care was limited). The main consequence of limited information sharing and uptake – apart from the delegation of responsibilities to the patient (repeating information and transferring test results between levels) [23, 26, 55] – was that it negatively affected the continuity of clinical management. For example, communication breakdowns led to unnecessary referrals and repetitions of medical tests, as well as the prescription of incompatible drugs. Previous literature has also cited medication errors [16] as well as delays in receiving care [16, 23, 56]. Thus, results also make manifest the interrelation of continuity types [4, 12] and, to a lesser degree, the relationship of continuity of care, an organization’s intermediate objective, with some of the organization’s final outcomes (quality of care, efficiency of health care delivery) [5, 57].

### Study limitations

Four limitations to this study warrant consideration. Firstly, patients with different profiles in terms of age, sex, and country of origin composed the study sample to gain variation in the discourse, and patients received care from diverse specialities as they suffered from different health conditions. The variation in the sample may have resulted in limited in-depth analysis. Nevertheless, we consider that the main themes were identified and that results were consistent given their concordance with a previous study conducted in Catalonia with COPD patients [34]. Analysis of continuity of care through segmenting data by different profiles and health services used could provide additional relevant results and should be conducted in future research, in order to fully understand any possible differences in patients’ perceptions. Secondly, three study areas were selected with different services management models to embody different healthcare contexts, which however do not take in, and hence might not represent, the whole spectrum. Nevertheless, they represent an important part of healthcare settings in the Catalan NHS. Thirdly, patients might possess limited knowledge on the different care coordination mechanisms implemented or the functioning of the healthcare organization or system in general. We wanted to analyse in depth how patients experience and perceive the phenomenon of continuity of care and describe ‘their reality’ by adopting a phenomenological approach. Our results show a great deal of identified implemented care coordination mechanisms, which further differed according to the study areas as noticed by the patients, in particular in terms of the information system implemented. In addition, findings are consistent when triangulating with health professionals’ perceptions [34] and care coordination indicators [37]. Fourthly, the lack of previous studies on continuity of care across levels, particularly regarding influencing factors and outcomes, makes it difficult to contrast and discuss our results.

### Recommendations for healthcare organizations

Patients largely perceived adequate continuity due to the implementation of a model of care based on primary health care with clear definition of roles, the introduction of care coordination mechanisms in their organization (especially EMR, but also mechanisms for informal communication and clinical meetings) and the physicians’ willingness to collaborate and use these mechanisms. The elements of discontinuity identified in this study (such as inconsistencies of treatment received from both care levels, missing or delayed referrals to secondary care or excessive waiting times for specific tests) serve to indicate where is room for improvement, and the factors influencing continuity (such as implemented care coordination mechanisms, appropriate conditions to use these mechanisms or the physicians’ willingness to collaborate and commitment to patient care) offer valuable insights to managers and professionals of healthcare organizations in these and other contexts on where to direct their care coordination efforts; which supposedly would also enhance the patient’s experience of a smooth trajectory. As postulated by different authors in literature on care coordination, and in agreement with our results, health managers should firstly stimulate formal and informal communication and collaboration in managing patient care (for example, by co-locating secondary care physicians at the primary care centre [58] or organizing regular meetings between professionals of the two care levels [58, 59]); and secondly, provide physicians with the necessary resources to use the coordination mechanisms put in place [27, 60, 61].

## Conclusions

This study shows that patients across the three study areas in Catalonia generally perceived that continuity of care across levels of care existed. However, patients also identified some interruptions, such as the unnecessary duplication of medical tests, long waiting times for specific tests, or the lack of use of clinical information, with some perceived negative consequences on quality of care and patient health. Patients linked (dis)continuity to certain factors related to the health system (clear distribution of roles), health services organizations (care coordination mechanisms such a shared EMR system and clinical meeting of physicians of both levels of care, co-location and insufficient resources) and physicians (willingness to collaborate, commitment to patient care, and the PCP’s technical competence). These could be addressed by managers and professionals of healthcare organizations in these and other contexts when aiming to improve services to achieve integrated care delivery according to patients’ actual healthcare needs. However, more research on continuity of care is needed; firstly, on perceived influencing factors in different contexts and secondly, on perceived consequences, in order to understand its full potential for improving quality of care and patient health.

## Additional files

Additional file 1: Interview guide for the analysis of continuity of care. (PDF 43 kb) Additional file 2: Subcategories and additional quotations. (PDF 68 kb) Additional file 3: Original Spanish language version of the quotations. (PDF 76 kb)

## Abbreviations

A&E: Accident and emergency department
COPD: Chronic obstructive pulmonary disease
EMR: Electronic medical records
GP: General practitioner
NHS: National health system
PCP: Primary care physician

## References

[1] Bodenheimer T (2008). Coordinating care: a perilous journey through the health care system. N Engl J Med.

[2] Ovretveit J (2009). Does improving quality save money? A review of evidence of which improvements to quality reduce costs to health service providers.

[3] McKee M, Nolte E (2009). Performance measurement for health system improvement: experiences, challenges and prospects. Chronic care.

[4] Reid RJ, Haggerty JL, McKendry R (2002). Defusing the confusion: concepts and measures of continuity of healthcare.

[5] Vázquez ML, Vargas I, Unger JP, Mogollón AS, Silva MRF, De Paepe P (2009). Integrated health care networks in Latin America: toward a conceptual framework for analysis. Rev Panam Salud Publica.

[6] Haggerty JL, Roberge D, Freeman GK, Beaulieu C (2013). Experienced continuity of care when patients see multiple clinicians: a qualitative metasummary. Ann Fam Med.

[7] O’Malley AS, Cunningham PJ (2009). Patient experiences with coordination of care: the benefit of continuity and primary care physician as referral source. J Gen Intern Med.

[8] Uijen AA, Schers HJ, van Weel C (2010). Continuity of care preferably measured from the patients’ perspective. J Clin Epidemiol.

[9] Haggerty JL, Reid RJ, Freeman GK, Starfield B, Adair CE, McKendry R (2003). Continuity of care: a multidisciplinary review. Br Med J.

[10] Wierdsma A, Mulder C, de Vries S, Sytema S (2009). Reconstructing continuity of care in mental health services: a multilevel conceptual framework. J Health Serv Res Policy.

[11] Parker G, Corden A, Heaton J (2011). Experiences of and influences on continuity of care for service users and carers: synthesis of evidence from a research programme. Health Soc Care Community.

[12] Waibel S, Henao D, Aller MB, Vargas I, Vázquez ML (2012). What do we know about patients’ perceptions of continuity of care? A meta-synthesis of qualitative studies. Int J Qual Health Care.

[13] Sparbel KJ, Anderson MA (2000). Integrated literature review of continuity of care. Part 1: conceptual issues. J Nurs Scholarsh.

[14] Freeman GK, Hughes J (2010). Continuity of care and the patient experience.

[15] Uijen AA, Schers HJ, Schellevis FG, van den Bosch WJ (2012). How unique is continuity of care? A review of continuity and related concepts. Fam Pract.

[16] Cowie L, Morgan M, White P, Gulliford MC (2009). Experience of continuity of care of patients with multiple long-term conditions in England. J Health Serv Res Policy.

[17] Aller MB, Vargas I, Waibel S, Coderch J, Sánchez-Pérez I, Llopart JR (2013). Factors associated to experienced continuity of care between primary and outpatient secondary care in the Catalan public healthcare system. Gac Sanit.

[18] Aller MB, Vargas I, Waibel S, Coderch J, Sánchez-Pérez I, Colomes L (2013). A comprehensive analysis of patients’ perceptions of continuity of care and their associated factors. Int J Qual Health Care.

[19] Uijen AA, Schellevis FG, van den Bosch WJ, Mokkink HG, van Weel C, Schers HJ (2011). Nijmegen Continuity Questionnaire: development and testing of a questionnaire that measures continuity of care. J Clin Epidemiol.

[20] Saltman RB, Rico A, Boerma W (2006). Primary Care in the Driver’s Seat? Organizational Reform in European Primary Care.

[21] Deborah TJ, Osheroff W (2008). Patient perceptions of inter-provider coordination of care. Hawaii Med J.

[22] Haggerty JL, Pineault R, Beaulieu MD, Brunelle Y, Gauthier J, Goulet F (2008). Practice features associated with patient-reported accessibility, continuity, and coordination of primary health care. Ann Fam Med.

[23] Nair KM, Dolovich LR, Ciliska DK, Lee HN (2005). The perception of continuity of care from the perspective of patients with diabetes. Fam Med.

[24] Miller AR, Condin CJ, McKellin WH, Shaw N, Klassen AF, Sheps S (2009). Continuity of care for children with complex chronic health conditions: parents’ perspectives. BMC Health Serv Res.

[25] van Walraven C, Oake N, Jennings A, Forster AJ (2010). The association between continuity of care and outcomes: a systematic and critical review. J Eval Clin Pract.

[26] Jones IR, Ahmed N, Catty J, McLaren S, Rose D, Wykes T (2009). Illness careers and continuity of care in mental health services: a qualitative study of service users and carers. Soc Sci Med.

[27] Vargas I, Mogollón AS, De Paepe P, da Silva MRF, Unger JP, Vázquez ML (2015). Do existing mechanisms contribute to improvements in care coordination across levels of care in health services networks? Opinions of the health personnel in Colombia and Brazil. BMC Health Serv Res.

[28] Van Servellen G, Fongwa B, Mockus D’Errico E (2006). Continuity of care and quality care outcomes for people experiencing chronic conditions: a literature review. Nurs Health Sci.

[29] García-Armesto S, Abadía-Taira MB, Durán A, Hernández-Quevedo C, Bernal-Delgado E (2010). Spain: health systems review. Health Syst Transit.

[30] Ministerio de Sanidad y Consumo. Marco estratégico para la mejora de la atención primaria en España: 2007-2012. Proyecto AP-21. Madrid: Ministerio de Sanidad y Consumo. Gobierno de España; 2007.

[31] Ley 14/1986 de 25 de abril, General de Sanidad. 26-4-1986. Boletín Oficial de Estado (BOE), n° 102.

[32] Decret 196/2010 de 14 de desembre, del sistema sanitari integral d'utilització pública de Catalunya (SISCAT). 16-12-2010. Diari Oficial de la Generalitat de Catalunya (DOGC), N° 5776.

[33] Vázquez ML, Vargas I (2009). Integrated healthcare organizations: a case study.

[34] Waibel S, Vargas I, Aller MB, Gusmão R, Henao D, Vázquez ML (2015). The performance of integrated health care networks in continuity of care: a qualitative multiple case study of COPD patients. Int J Integr Care.

[35] Departament de Salut, Servei Català de la Salut. La veu de la ciutadania: com la percepció de la ciutadania es vincula a la millora dels serveis sanitaris i el sistema de salut de Catalunya. Barcelona: Department de Salut; 2015.

[36] Bartoll X, Salvador M, Allué N, Borrell C (2011). Enquesta de salut de Barcelona 2011.

[37] Aller MB, Vargas I, Coderch J, Calero S, Cots F, Abizanda M (2015). Development and testing of indicators to measure coordination of clinical information and management across levels of care. BMC Health Serv Res.

[38] Reeves S, Albert M, Kuper A, Hodges BD (2008). Why use theories in qualitative research?. BMJ.

[39] Kuper A, Lingard L, Levinson W (2008). Critically appraising qualitative research. BMJ.

[40] Pope C, Ziebland S, Mays N (2000). Qualitative research in health care. Analysing qualitative data. BMJ.

[41] Walsh J, Harrison JD, Young JM, Butow PN, Solomon MJ, Masya L (2010). What are the current barriers to effective cancer care coordination? A qualitative study. BMC Health Serv Res.

[42] Tarrant C, Windridge K, Baker R, Freeman G, Boulton M (2015). ‘Falling through gaps’: primary care patients’ accounts of breakdowns in experienced continuity of care. Fam Pract.

[43] Walker KO, Labat A, Choi J, Schmittdiel J, Stewart AL, Grumbach K (2013). Patient perceptions of integrated care: confused by the term, clear on the concept. Int J Integr Care.

[44] Kringos DS, Boerma WG, Hutchinson A, Van der Zee J, Groenewegen PP (2010). The breadth of primary care: a systematic literature review of its core dimensions. BMC Health Serv Res.

[45] Wodskou PM, Host D, Godtfredsen NS, Frolich A (2014). A qualitative study of integrated care from the perspectives of patients with chronic obstructive pulmonary disease and their relatives. BMC Health Serv Res.

[46] Barnet M, Shaw T (2013). What do consumers see as important in the continuity of their care?. Support Care Cancer.

[47] Preston C, Cheater F, Baker R, Hearnshaw H (1999). Left in limbo: patients’ views on care across the primary/secondary interface. Qual Health Care.

[48] Gulliford M, Cowie L, Morgan M (2011). Relational and management continuity survey in patients with multiple long-term conditions. J Health Serv Res Policy.

[49] Lin CY (2012). Improving care coordination in the specialty referral process between primary and specialty care. N C Med J.

[50] Breton M, Haggerty J, Roberge D, Freeman GK (2012). Management continuity in local health networks. Int J Integr Care.

[51] Grumbach K, Selby JV, Damberg C, Bindman AB, Quesenberry C, Truman A (1999). Resolving the gatekeeper conundrum: what patients value in primary care and referrals to specialists. JAMA.

[52] Abásolo I, Negrin-Hernandez MA, Pinilla J (2014). Equity in specialist waiting times by socioeconomic groups: evidence from Spain. Eur J Health Econ.

[53] Spanish Government. Programa Nacional de Reformas. Madrid: La Moncloa; 2012.

[54] Michiels E, Deschepper R, Van der Kelen G, Bernheim JL, Mortier F, Vander SR (2007). The role of general practitioners in continuity of care at the end of life: a qualitative study of terminally ill patients and their next of kin. Palliat Med.

[55] O’Cathain A, Coleman P, Nicholl J (2008). Characteristics of the emergency and urgent care system important to patients: a qualitative study. J Health Serv Res Policy.

[56] Golden SL, Nageswaran S (2012). Caregiver voices: coordinating care for children with complex chronic conditions. Clin Pediatr.

[57] Gröne O, Garcia-Barbero M (2002). Trends in Integrated Care: Reflections on Conceptual Issues (EUR/02/5037864).

[58] Zuchowski JL, Rose DE, Hamilton AB, Stockdale SE, Meredith LS, Yano EM (2015). Challenges in referral communication between VHA primary care and specialty care. J Gen Intern Med.

[59] Berendsen AJ, Benneker WH, Meyboom-de Jong B, Klazinga NS, Schuling J (2007). Motives and preferences of general practitioners for new collaboration models with medical specialists: a qualitative study. BMC Health Serv Res.

[60] van Wijngaarden JD, de Bont AA, Huijsman R (2006). Learning to cross boundaries: the integration of a health network to deliver seamless care. Health Policy.

[61] Havens DS, Vasey J, Gittell JH, Lin WT (2010). Relational coordination among nurses and other providers: impact on the quality of patient care. J Nurs Manag.

[62] Servei Català de la Salut (2010). Registro Central de Asegurado.

