# Supplementary material for: Continuity of clinical management and information across care levels: perceptions of users of different healthcare areas in the Catalan national health system
Source: BMC Health Serv Res. 2016 Sep 2;16(1):466. doi: 10.1186/s12913-016-1696-8 (PMC5010665; doi:10.1186/s12913-016-1696-8)
Supplement: Additional file 1: — Interview guide for the analysis of continuity of care. (PDF 43 kb) [file 12913_2016_1696_MOESM1_ESM.pdf]

Additional file 1 - Interview guide for the analysis of continuity of care (translated from Spanish)

Date of interview:
Time of initiation/finalization:
Healthcare organization:
Location:

Contextual analysis and socioeconomic analysis: sex, age, date of birth, place of birth / time in Catalonia / Spain, profession, occupation, level of education, private health insurance;

CONTEXTUALIZATION AND EXPERIENCE WITH THE USE OF HEALTHCARE SERVICES

Health condition

How do you feel / how is your health?

Use of healthcare services

What type of health services have you used? For what kind of disease / condition? How was your experience with the health services used?

CONTINUITY OF CLINICAL MANAGEMENT ACROSS CARE LEVELS

Transition between PC ↔ SC[1]

*Has your GP referred you once to a **specialist**? Why? How was it?*

*Has your specialist referred you once to your **GP**? Why? How was it?*

Have you ever been hospitalized? Have you gone to the emergency room once? Why? After hospital/emergency discharge, have you been sent to a different service? Why? How was it?

Accessibility to PC and SC

*Do you consider that your GP/ specialist is **accessible**? Why?*
*What **facilitates** the use of healthcare services? Why?*
*What **difficulties** do you encounter when you try to use healthcare services? What do you do to overcome those difficulties?*

General perception of coordination and collaboration

*Do you think that the **care is coordinated** among the physicians that see you? Why? Do you consider that the physicians **work together**? Why?*

Care coherence (tests, indications, treatment)

*Do the physicians recommend you the **same treatment**? Have you ever received **conflicting information**? What information? What did you do in this case?*

*When you are attended to at a different service, do physicians **repeat diagnostic tests**? Why? What do you think about it?*

---

[1] PC - primary care; SC – secondary care (outpatient care, emergency care and inpatient care)

CONTINUITY OF INFORMATON ACROSS CARE LEVELS

Information transfer across care levels and its use

Do you think that the GP ***knows the medical indications*** *that the specialist has given you previously, before you explain them to the GP? And the specialist knows those of the GP? Why? What do you think about it?*

Have you had to ***repeat*** *your clinical history or test results during the visits with your GP / specialist? Why? And in the emergency department / hospital?*

RELATIONAL CONTINUITY (INTRA-LEVEL)

Person responsible for care provision

Do you think that there is someone who is ***responsible*** *for your care (treatment)? Who is it? Why?*

Is there any ***other professional*** *(nurse, social worker, etc.) that you consider important in your care? Why*

Consistency of clinical personnel

Are you always ***seen by the same*** *GP / specialist? Why? What do you think?*

Patient-physician relationship

How is the ***relationship*** *with your GP / specialist / other professionals identified? (Do you think the physician* ***cares*** *about you? Why? Do you* ***trust*** *your physician? Why?)*

Knowledge about values and preferences

Do you think that your GP / specialist ***knows you****? Why? Does he/she knows your* ***clinical history*** *(antecedents, medication, tests performed)* ***personal circumstances****,* ***needs****? Why? What do you think?*

Patient-physician communication

How is the ***communication*** *between you and your GP / specialist? Does the physician* ***understand*** *you? And do you understand your physician? What do you think about the* ***information*** *that your GP / specialist gives you?*
