# Supplementary material for: Continuity of clinical management and information across care levels: perceptions of users of different healthcare areas in the Catalan national health system
Source: BMC Health Serv Res. 2016 Sep 2;16(1):466. doi: 10.1186/s12913-016-1696-8 (PMC5010665; doi:10.1186/s12913-016-1696-8)
Supplement: Additional file 2: — Subcategories and additional quotations. (PDF 68 kb) [file 12913_2016_1696_MOESM2_ESM.pdf]

Additional file 2 – Subcategories and additional quotations

A. Perception of the existence of (dis)continuity of clinical management and information across care levels

Dimension and subcategories	Quotation
**Consistent care across levels**	
Consistency in diagnosis, treatment and medical recommendations without conflicts	*(The GP) saw me and said "Well, this looks like a hernia to me" (…) He sent me to a surgeon and she said the same thing, that I had a hernia and that, well, I had to have an operation. (Baix Empordà, male patient, 46)*
	What they recommend is always the same, for me, personally, "you've got to lose weight, you've got to do more exercise, and basically watch what you eat". They tell me the same thing here, there and everywhere. (…) For now I haven't come across any contradictions or controversies. (Barcelona, male patient, 48)
	The emergency doctor told her (my wife) that it was nothing and it wasn't worth going to A&E for, that she should go to her normal doctor on Monday, and when we went to the doctor on Monday he said "this isn't right", that she had this, this and this problem, that it was an emergency and that they had to attend to her. (Baix Empordà, male patient, 63)
	A year ago I was in limbo (…) my GP says: "I don't think there's anything wrong with you, you don't have any trace of arthritis. It doesn't look that way", (…) the orthopaedic specialist says that I do have a bit of arthritis (…) you feel a bit like you're alone (…) so, I don't see much communication of a direct, more sincere, more open kind (…) everyone goes their own way. (Barcelona, male patient, 45)
Repetition of medical tests only when necessary	*No (no duplication). The only thing - and actually I felt they were right to do it again and besides it's a cheap and easy test to do - was a urine test (…) The urologist thought it would be wise to do the urine test again, I imagine to make certain there was blood in it, I think that makes perfect sense (…) I didn't have to do another blood test or anything, just the urine test. (Barcelona, female patient, 52)*
	The doctor requested a blood test for me (…) In the (primary care) health centre they take a long time to give you the results, well, a week. Then I had another four days of fever and so I decided to go to emergencies (…) and there they do blood tests, urine tests and everything really quickly (…) I felt bad that my GP had already done it once. (Girona, female patient, 26)
Adequate follow-up across care levels	*It was my GP that saved me (…) when she saw me she sent me straight to the hospital. In all truth it's my GP who's always referred me to all the different services. (Baix Empordà, female patient, 41)*
	I think that all the doctors coordinate with each other, in principle yes, I do believe that, that the doctors all know about the rest of their colleagues (…) They all know each other and they say: "go and see such and such, who can treat this really well" or (…) "you'd be better off seeing this doctor, she knows more about this than me" and they refer you to another doctor. (Baix Empordà, male patient, 61)

	For me there certainly was (communication), for example I went to the doctor and told her I wanted to see the gynaecologist; so she sent a message on her computer and she told me: "you've got an appointment for this date". Then I went to the gynaecologist and she said "yes, Dr (name) got in touch with me asking me to see you" (Baix Empordà, female patient, 70).<br>*Every time I've been, I've been well attended to. (…) I go in; I explain what problems I'm having. If she thinks she can solve it herself, she prescribes me something and if not, she refers me to the hospital. If it's something, you know, more specialized, then she sends me to the hospital. (Barcelona, male patient, 40)*<br>*When you have an operation, they give you the results, they give you the paperwork, so I take those papers and show them, I showed them to my doctor (GP) so she could see them, and see what the situation was, and was up to date with everything that was happening. (Baix Empordà, female patient, 39)*<br>*Every time I see a specialist, they send me back to my GP afterwards with a report of what they told me, at the end of the day it's my GP who makes out the prescriptions, it's him who has to be well informed of everything, of every detail. (Barcelona, male patient, 48)*<br>*He (the cardiologist) monitors everything for you, or he sends you, for example, if he has to send you for tests, he sends you to the GP to have them done. (…) "Well bring me more test results in a years' time". And I have to go and ask my GP for them. (Barcelona, male patient, 70)*
**Accessibility across care levels**	
Timeliness of secondary care visits after referral from primary care	*If it's urgent, it's fast, really – because when they found the thing with my eye (…) it was a Friday, and by Wednesday I was seeing the ophthalmologist, so it really is quick. When I came here to emergencies (at the primary care centre) they sent me over to the orthopaedic department in no time, that was four days too – urgent. (Baix Empordà, female patient, 41)*<br>*My GP, when she saw it was serious she sent me to emergencies (…) she referred me straight away. (Barcelona, female patient, 53)*<br>*10-15 days (for the lung specialist), so that seems alright to me, pretty good really. Actually, I think (…) the gynaecologist was even quicker than that, they gave me an appointment for, like, the same week (…) I found it pretty amazing (…) It looked like a pretty fast service to me from one doctor to another, for all of them - the gynaecologist, the ENT specialist, the orthopaedic surgeon... (Barcelona, female patient, 28)*<br>*If they see that it's serious they say yes, to the orthopaedic department, but if not, no, because they say it takes ages. I don't know, I broke three toes (…) my GP did the X-rays and everything and he said: "right, well if something goes wrong than we'll send you to the orthopaedic department, but if not, we won't", and I didn't go in the end. (Barcelona, female patient, 26)*<br>*A few months ago I had this problem with my saliva gland, it was all swollen. It was nothing, but the doctor asked for an ultrasound to make sure. Well they gave me an appointment for the ultrasound for four weeks down the line. Why four weeks? Because the (primary) health centre doesn't do ultrasounds, they're done at the Hospital del Mar, and the Hospital del Mar is completely inundated (…) the endoscopy was done in ten days though, so I suppose it's down to each department how quickly they can go and that one was done really quickly. (Barcelona, male patient, 54)*

	Well for the specialist it is far too long to wait, yes, I mean…with specialists it's a problem, it's a real problem. Yeah, because of course the specialists take ages. (Girona, female patient, 55)<br>*When the doctor (ophthalmologist) saw me she said 'we need to do a cataract operation'. So the whole process got underway, they did all the tests, and then she said 'we'll call you' and that's as far as we've got (…) I guess they must have a lot of people waiting and of course they'll be calling the most urgent cases first (…) It would be great to have had it done already, of course, the longer it takes the worse it gets. (Girona, male patient, 64)*
Timeliness of primary care appointments after having tests performed	*I went for an X-ray and two days later I had my appointment (with the GP), I guess maybe they had a free slot somewhere. In fact they do always check to see if there's a free slot, or maybe someone had an appointment and couldn't make it for some reason, so they always try to squeeze you in. (Baix Empordà, male patient, 40)*<br>*I asked my doctor for an appointment with the ophthalmologist; oh my god, yes – not till next month (…) the ophthalmologist, I imagine but I don't know for sure, will have to do some tests or whatever and those tests will have to go to my GP. And then I'll have to go to the GP so she can tell me what the results are, I guess. That'll be another month. About three months or so just to find out what's wrong with my eyes, yup. (Barcelona, female patient, 29)*
**Information sharing across levels and its use**	
Information transfer via computer across care levels	*She (the GP) already had the information (from A&E), oh yes, it's all very well coordinated. Yes, they know what's going on in emergencies at any given time and they know everything (…) In emergencies they have my records, as everything is coordinated via the computer, they know everything in an instant, it's really very good. (Baix Empordà, female patient, 43)*<br>*All the doctors here are connected and each one adds their notes (…) I don't have to tell them "My back hurts. I've got a dodgy kidney". No. (The doctor) reads the report and translates: "So you've got this, this and this, OK so where does it hurt?" And that way they can get the appointment going quickly. (Baix Empordà, male patient, 61)*<br>*The (PCP) has all the results of the tests the specialists did because he can see the results on the computer now, can't he? (…) everything is sent by computer, that's how they communicate. (Girona, female patient, 66)*<br>*I think the communication by computer is pretty good actually. Like when I last went to the Hospital del Mar, as soon as I walked in the door, the guy was telling me what was wrong with me (…) I think that the information does go from lower to higher (primary to specialist care) but I don't think it goes back the other way. (Barcelona, female patient, 28)*
Patient transferring information across care levels	*Sometimes if I go to the cardiologist and he changes one of my pills then he gives me a piece of paper and I take it to my GP who is the one who makes out the prescriptions. And so she puts it into the computer and then they all know. Oh yes, it's all very coordinated and works well. (Barcelona, male patient, 70)*<br>*Yes (they share information), because I took (the PCP) what they gave me in Drassanes (A&E). (Barcelona, female patient, 37)*<br>*When I was discharged from the Hospital del Mar they gave me all the paperwork, everything, all of it, they gave it to me. And that information, my GP (entered it) into the computer and there it all is, everything. (Barcelona, female patient, 53)*

	When I go (to the PCP) I never have to take any papers (from the specialist) with me because they automatically look it up and all the information is there and that's it. (Baix Empordà, female patient, 46)
Uptake of information from medical records	*When I went to the ophthalmologist, they were checking all the time on the medication I was taking, and the whole report that my GP had sent. (Baix Empordà, male patient, 63)*
	Whoever comes in, they've got all their records, they can see all of them. And then they check on the computer, they see the last appointments you've had, and they already know what's wrong with you. (…) My normal doctor, whenever I walk in, she's already read up on it, she already knows why I'm there. When I go in she's already got the picture. That's also really good. (Girona, female patient, 55).
	Every time I go it's a different person (PCP) because he's on sick leave (…) It's a bit of a mess. I have to tell it all over again every time. (…) They can see it (clinical history) but… I don't know… if they had to read through all of it…I don't know, it's quicker just to tell them. (Barcelona, female patient, 26)

B. Factors influencing (dis)continuity of clinical management and information across care levels

Category	Quotation
**Factors related to the health system**	
Distribution of roles between physicians of the two care levels	*GPs stay on top of any chronic illnesses you have, don't they? And then after seeing how it's going, if we've had any kind of relapse (…) And once they send us to the specialist, that issue has now been, how should I say, dealt with; they put it aside. And they handle different matters when you come to them with health problems. (Girona, male patient, 42)*
	Each doctor prescribes what they need to prescribe in their speciality, and the GP is the one who has the full story and, well…if they have any recommendations to make then they make them. (Barcelona, male patient, 45)
	The cardiologist is the one who monitors us at regular intervals; the appointment is already programmed to have that followed up. And the GP only deals with the things that come up (for my mother) on a day to day basis. If she has an infection, if she's got 'flu or a cold (…) The GP has to monitor all that constantly. (Girona, female patient, 82
	My perception is this: that (GPs) want to avoid sending you to the specialist (…) now it's as if (the GP) wants to solve problems without referring you to the specialist. So, I think that's a field that's too big for them, for the GPs, because a jack of all trades is a master of none (Girona, male patient, 47)
**Factors related to the health services organizations**	
Insufficient resources	*The ones in emergencies, I think they must be overloaded with work and maybe they're short of staff. (…) I don't know if they make a report on every person that comes in. I doubt they have the time, because they're pretty inundated with people. (Baix Empordà, female patient, 42)*
	I feel that, well, yes, that my GP and the specialists….well, I haven't noticed any real lack of coordination, really. For me, yes, it's worked well, I've felt well attended to (…) And of course these cuts, all they're going to lead to is a breakdown in this coordination. (Barcelona, female patient, 52)

	The specialist doctors, like the ophthalmologist for example, there aren't enough of them for the number of people there are around here. Normally if you ask for an appointment with the eye doctor you'll get one for six months' time, it's crazy. (Barcelona, male patient, 54)
Care coordination mechanisms	*Duplicate my tests? No, quite the opposite, as everything is on the computer: "OK well I won't take another blood test because you had one a month ago." (Baix Empordà, female patient, 41)*<br>*I don't know when they digitalised all of this, but they hold a lot of information now (…) analyses and tests (…) I think that the coordination between the doctors is the computer (Baix Empordà, female patient, 52)*<br>*I know they have meetings because when I went to the orthopaedic specialist, he said to me: "no, we discussed your case in last Thursday or Friday's meeting" (…) and my GP told me "the physio told me that instead of having to go and see him, that I should change this medication for you", so really I think yes, there is coordination. (Baix Empordà, male patient, 41)*<br>*I know there are meetings (…) I mean they all know each other and they talk about cases and…yes, (…) it's lucky really, because that way you don't get sent to a specialist and the specialist doesn't even know who the GP or doctor is that sent you. (Baix Empordà, male patient, 41)*<br>*They also talk to each other from time to time. Sometimes I'm talking to my doctor and she says: "right, yes, I've spoken to such and such, I've spoken to this person, and that person, and we think this is what's going on", they talk to each other (….) and then the results, you find out what's wrong straight away, they don't make you wait. (Baix Empordà, male patient, 61)*<br>*Now with this thyroid thing, the doctor says 'it looks like it's this to me but I'm going to consult with the endocrinologist' and she made enquiries taking into account that I'd already had this illness before. (…) On my next visit she told me she'd sent an email to the other doctor and that she had replied, but in quite a reasonable time frame, really. (Girona, female patient, 55)*
Co-location of physicians	*Because it's all in the same health centre, the results are right here on the computer. The computers are connected within the health centre (…) the notes that (the ENT specialist) makes, she puts them into the computer (…) when I go to the GP he looks on the computer and says "right, yes, I can see her notes here". (Barcelona, male patient, 48)*<br>*The physio (at the primary care centre) asked me: "hey, are you OK?" And I said "how did you know that about me?" (…) "because I can see it all here (…) your GP and I can both see it." (Barcelona, female patient, 26)*
**Factors related to the physicians**	
Willingness to collaborate	*If you go to the specialist and the doctor has already done some kind of test like an X-ray or something, he tells you to take it with you (…) to give to the specialist (…) so it doesn't have to be done all over again. (Girona, female patient, 64)*<br>*The GP, in principle (…) wanted me to have (blood) tests done a couple of times a year. But as I'm having them done now at the Hospital del Mar, well all I have to do is take him the results, so I don't have to have the same tests twice, because they're checking on exactly the same thing. (Barcelona, male patient, 48)*

Commitment to the patient care	*The specialist is the one who supposedly has to delve more deeply into the problem (…) it's the specialists (…) who should get together with the other doctors and investigate together, shouldn't they? (…) I can't see any interest in actually solving the problem, but rather, how should I put it, to keep things moving for now. So the problem is such and such? Well we'll just give him this and that's dealt with." (Girona, male patient, 42 years)*<br>*I'm just another number to her (PCP), that's all. That's how I feel (…) because when I went with my leg problem, I didn't feel... that she was even vaguely interested, like, in finding out whether there was an earlier slot available, you know? She just said straight off: "no", just like that (…) because I asked if they could get me an appointment for December, because I wanted to know what was wrong with my leg, you know? (Barcelona, female patient, 37)*
Primary care physician's technical competence	*If I have a particular problem, I don't know exactly what the problem is. So I don't know if it's a case of going to the cardiologist or the orthopaedic department, let's say. I don't know where I need to go (…) Someone has to steer this thing, and that person is the GP. (Barcelona, male patient, 55)*<br>*The GP is the one who assesses you and decides whether or not it's really necessary for you to see (the specialist). (Baix Empordà, female patient, 46)*
