# Supplementary material for: Continuity of clinical management and information across care levels: perceptions of users of different healthcare areas in the Catalan national health system
Source: BMC Health Serv Res. 2016 Sep 2;16(1):466. doi: 10.1186/s12913-016-1696-8 (PMC5010665; doi:10.1186/s12913-016-1696-8)
Supplement: Additional file 3: — Original Spanish language version of the quotations. (PDF 76 kb) [file 12913_2016_1696_MOESM3_ESM.pdf]

Additional file 3 – Original Spanish language version of the quotations

A. Perception of the existence of (dis)continuity of clinical management and information across care levels

Dimension and subcategories	Quotation
**Consistent care across levels**	
Consistency in diagnosis, treatment and medical recommendations without conflicts	*Miran que sea compatible una medicación con otra. Al tomar el (acenocumarol), este también, es complicadísimo para la medicación. Muchos medicamentos no son compatibles con el (acenocumarol) y tienen que estar siempre mirando todo eso. (Barcelona, hombre, 70 años)*
	(El médico de cabecera) me vio y me dijo "Bueno, me parece que esto es una hernia" (…) Me mandó a una cirujana y me dijo lo mismo, que tenía una hernia y bueno que me tenían que operar. (Baix Empordà, hombre, 46 años)
	Lo que aconsejan es lo mismo, personalmente, "tienes que perder peso, tienes que hacer más ejercicio, y básicamente pues controlar los alimentos". Me dicen lo mismo en un sitio, en el otro y en el otro. (…) Contradicciones o controversias hasta ahora no he encontrado con ninguno. (Barcelona, hombre, 48 años)
	El médico de urgencias le dijo (a mi mujer) que no tenía nada y que no valía la pena ir a urgencias, que el lunes fuera al médico normal, y cuando el lunes fuimos al médico me dijo "esto no puede ser", que tenía esto, esto y esto, que era una urgencia y que le tenían que atender. (Baix Empordà, hombre, 63 años)
	Hace un año se creó un limbo (…) dice mi médico de cabecera: "me parece que tú no tienes nada, tú no tienes nada de artrosis. Me parecía que no", (…) el traumatólogo especialista dice que tengo un poco de artrosis (…) te sientes un poco solo (…) entonces, yo veo que no hay una comunicación directa, más sincera, más abierta (…) cada uno hace lo suyo. (Barcelona, hombre, 45 años)
Repetition of medical tests only when necessary	*Yo aquí vendré a urgencias (en el CAP) ¿no? y me haré una prueba (…) y si voy a Palamós allí, me hacen las mismas. Allí quieren ver las pruebas estas, claro porque quieren saberlo si es así (…) Bien, claro que se asegure bien el especialista de lo que tiene que hacer. (Baix Empordà, hombre, 75 años)*
	No (no duplicación). La única cosa que además yo encontré que estaba bien que lo hiciesen de nuevo, y además que es una prueba que es barata, que se hace fácil, que fue una prueba de orina (…) El urólogo encontró conveniente volver a hacer una prueba de orina, me imagino para cerciorarse de nuevo de que había sangre, yo lo encuentro lógico. (…) No me volvió a mandar a hacer una analítica de sangre y eso, solamente la prueba de orina. (Barcelona, mujer, 52 años)
	El médico me pidió el análisis de sangre (…) En el CAP tardan mucho en darte los resultados, bueno, una semana. Entonces yo llevaba cuatro días más con fiebre y decidí ir a urgencias (…) allí te hacen el análisis de sangre, de orina y de todo súper rápido (…) Me supe mal que ya lo ha hecho el médico de cabecera. (Girona, mujer, 26 años)

Adequate follow-up across care levels	*La que me salvó fue la doctora de cabecera (…) cuando me vio me mandó directamente para el hospital. La verdad es que la doctora de cabecera es quien me ha ido derivando a todos los servicios. (Baix Empordà, mujer, 41 años)*
	Yo creo que los médicos están todos coordinados, en principio creo que sí, que los médicos cada uno sabe las cosas de los demás (…) Entre ellos se conocen y te dicen: "ves a ver a Fulano de tal, que para esto te tratará bien" o (…) mejor que vayas a la doctora tal, que ella te lo verá mejor que yo" y te derivan a otro médico. (Baix Empordà, hombre, 61 años)
	Conmigo ya te digo (había comunicación) por ejemplo yo fui a la doctora y le dije que quería ir a la ginecóloga; entonces ella le mandó por ordenador un mensaje y me dijo: "para tal día tienes visita". Luego fui a la ginecóloga y me dijo "sí, la doctora (nombre) se ha puesto en comunicación conmigo para que te visite" (Baix Empordà, mujer, 70 años).
	La verdad es que cuando yo percibo que el médico de cabecera no sabe bien bien por donde se mueve, por donde se encuentra y le pido que me derive a algún sitio, la verdad es que accede. (Girona, hombre, 47 años)
	Todas las veces que he ido, me ha atendido bien (…) Yo entro, le digo los problemas que tengo. Si ella considera que me lo puede solucionar ella, me receta lo que sea y si no, me deriva al hospital. Si es una cosa, no sé, más especializada pues me deriva al hospital. (Barcelona, hombre, 40 años)
	Cuando te operan, pues te dan los resultados, te dan los papeles, entonces estos papeles los traigo y se los enseño, los enseñé a la doctora (de cabecera) para que lo viera, y viera lo que hay, y esté al corriente de todo lo que pasa. (Baix Empordà, mujer, 39 años)
	De urgencias (…) pues te mandan para casa y te dicen: "pues mire, mañana vaya usted al médico de cabecera", porque claro, normalmente te tiene que visitar y te tiene que poner la medicina que te corresponda, el médico de cabecera. Ya hace muchos años que funciona muy bien. (Girona, mujer, 66 años)
	Cada vez que veo un especialista, después (me manda) al médico de cabecera con el informe de lo que me ha dicho, después de todo es el médico de cabecera él que hace las recetas, es él que tiene que estar informado de todo, de cada cosa. (Barcelona, hombre, 48 años)
	Él (cardiólogo) ya te lleva todo el control, o te manda, por ejemplo si te tiene que enviar a hacer análisis te manda a la de cabecera para que te los hagas. (…) "Pues al año que viene me traes un análisis". Y yo tengo que ir a pedírsela a la de la cabecera. (Barcelona, hombre, 70 años)

Accessibility across care levels

Timeliness of secondary care visits after referral from primary care	*Ha sido un tiempo prudente (…) quizá un mes o así, más o menos, pero como tampoco es nada importante yo considero que es un tiempo que está bien. (Baix Empordà, mujer, 46 años)*<br>*Si es urgente, es rápido, sinceramente porque cuando me encontraron lo del ojo (…) fue un viernes y el miércoles ya me estaba visitando el oculista, o sea es rápido. Cuando vine aquí a urgencias (del CAP) me mandaron a traumatología fue nada, también en cuatro días – urgente. (Baix Empordà, mujer, 41 años)*<br>*Mi médica de cabecera, cuando ella me vio grave me mandó a urgencias (…) en seguida me derivó. (Barcelona, mujer, 53 años)*<br>*10-15 días (para el neumólogo), o sea me parece bien, bastante correcto. Creo, incluso (…) el ginecólogo fue mucho más rápido, o sea me dieron casi para la misma semana. (…) Me pareció algo increíble (…) Me ha parecido un servicio bastante rápido entre un médico y otro, en todos, tanto en el ginecólogo, como en el otorrino, como en el traumatólogo. (Barcelona, mujer, 28 años)*<br>*Cuando vea si es grave o así entonces sí, traumatólogo, si no, no, porque dicen que va muy lento. No sé, me rompí tres dedos del pie (…) el médico de cabecera me hizo las radiografías y todo y dice: "bueno, si algo no va bien pues al traumatólogo, pero si no, no", y ya no fui al traumatólogo. (Barcelona, mujer, 26 años)*<br>*Hace unos meses tuve un problema que se me inflamó allí en la glándula esta salival. No era nada pero el médico para asegurarse me pidió una ecografía. Pues la ecografía me la dieron para cuatro semanas. ¿Por qué a las cuatro semanas? Porque la ecografía no la hace el CAP, la hace el Hospital del Mar y el Hospital del Mar está colapsado (…) la endoscopía me hicieron en diez días, o sea supongo que según que departamento tiene más agilidad o no y me la hicieron muy rápido. (Barcelona, hombre, 54 años)*<br>*Bueno, especialista sí que lo encuentro exagerado (el tiempo de espera), o sea, especialistas es ya un cierto problema, es un problema. Sí, porque claro, tardan, los especialistas tardan, tardan mucho. (Girona, mujer, 55 años)*<br>*Cuando me vio la doctora (oftalmóloga) me dijo 'tendríamos que operarte de cataratas'. Entonces empezó el proceso, me hicieron todo el estudio, y entonces ya me dijo ella 'ya te llamaremos' y hasta ahí (…) Se ve que hay muchas personas y claro, irán llamado a las que están más graves (…) Que yo ya quisiera estar operado, claro, cuando más tarde es peor. (Girona, hombre, 64 años)*<br>*Me dijeron (en la atención primaria) que la resonancia me lo iban a dar para fines de enero (…) como un mes más o menos, pero imagínate un mes, yo con mi pierna, con mucho dolor y no sabía que tenía ni nada ¿sabes? era también… era preocupante. (Barcelona, mujer, 37 años)*

Timeliness of primary care visits after having tests performed	*Fui a hacer la radiografía y a los dos días ya tenía la visita (con el médico de cabecera), supongo que a lo mejor tenían algún hueco. Eso sí, siempre miran que hay algún hueco, o alguien tenía vista y no podía ir por cualquier cosa, entonces te colocan siempre. (Baix Empordà, hombre, 40 años)*<br><br>*Al oftalmólogo pedí cita, con la doctora; ostras sí, hasta el mes que viene (…) el oftalmólogo, supongo porque no sé me hará unas pruebas, lo que sea y esas pruebas tendrán que ir a la de cabecera. Y luego yo tendré que ir a la de cabecera, que me diga cómo ha salido el resultado, supongo. Échale otro mes. Unos tres meses o así para saber lo que tengo en los ojos, sí. (Barcelona, mujer, 29 años)*<br><br>*La vuelta al médico de cabecera, bueno ya pides hora para el día siguiente ¿no? Cuando te dicen "a las cuatro semanas te van a hacer la ecografía" pues sabes que al día siguiente, a los dos días, pues pides hora para el médico de cabecera. (Barcelona, hombre, 54 años)*
**Information sharing across levels and its use**	
Information transfer via computer across care levels	*Ella (la médica de cabecera) ya tenía la información (de urgencias), sí, sí, está todo muy bien coordinado. Sí, ellos en cualquier momento saben lo que pasa en urgencias y saben todo (…) En urgencias tienen el historial mío, como está todo coordinado por el ordenador, en un momento saben todo, esto es muy bien. (Baix Empordà, mujer, 43 años)*<br><br>*Todos los médicos de aquí están conectados y cada uno pone sus cosas (…) No tengo que decirle: "Me duele la espalda. Tengo un riñón mal". No. (El médico) lee el informe y traduce: "usted tiene esto, esto y esto", vale ¿a ver que le duele? Y así pueden empezar la visita muy rápido. (Baix Empordà, hombre, 61 años)*<br><br>*Ahora a nivel de información, últimamente se ha informatizado bastante bien, y el médico de cabecera tiene también acceso a las pruebas que me hacen al hospital (…) que antes tenías que ir con una carpeta así (gordo) con las radiografías. (Girona, hombre, 79 años)*<br><br>*Él (médico de cabecera) tiene todos los resultados de las pruebas que me han hecho los especialistas porque ahora por ordenador él ve los resultados ¿no? (…) se transmiten todo por ordenador, por ahí se comunican. (Girona, mujer, 66 años)*<br><br>*Claro que la tienen (la información en urgencias…) porque si no la tuvieran es un peligro para el paciente. Te pongan, a lo mejor te inyecten algún medicamento que es contrario a lo que estás tomando. No, esto es sagrado. (Girona, mujer, 66 años)*<br><br>*Creo que tienen una comunicación por ordenador bastante buena, creo. Igual que cuando fui también ahora al Hospital del Mar, según entré por la puerta, el chico se estaba leyendo lo que me pasaba (…) Yo creo que la información sí que va de menos a más (atención primaria a especializada) pero no creo que haya vuelto. (Barcelona, mujer, 28 años)*

Patient transferring information across care levels	*A veces si yo mismo voy al cardiólogo y me cambia una pastilla y entonces me da un papelito y yo se lo llevo a la doctora de cabecera que es la que hace las recetas. Y ya lo introduce en el ordenador y ya lo saben. Sí, sí, eso está muy coordinado y bien. (Barcelona, hombre, 70 años)*<br>*Sí (comparten la información), porque yo llevé (al médico de cabecera) lo que me dieron en Drassanes (urgencias). (Barcelona, mujer, 37 años)*<br>*A mí cuando me dieron el alta del Hospital del Mar me dieron todas las hojas, todo, todo, todo, me dieron. Y esa información, mi médica de cabecera (la introdujo) al ordenador y ahí está, todo. (Barcelona, mujer, 53 años)*<br>*Siempre, yo cuando voy (al médico de cabecera) no tengo que llevar ningún papel (del especialista) porque ellos automáticamente miran y le salen toda la información y ya está. (Baix Empordà, mujer, 46 años)*
Uptake of information from medical records	*El oftalmólogo cuando fui, se miró la medicación que tomaba, todo el informe que le había puesto la doctora de cabecera en todo momento. (Baix Empordà, hombre, 63 años)*<br>*Venga el que venga todos tienen tu historial, todos se lo leen. Y a parte ellos miran en el ordenador, miran los últimos días que has ido, y ya saben lo que tienes. (…) La doctora normal que tengo, siempre cuando entro, ella ya se lo ha mirado, ya sabe por qué vengo. Cuando yo entro ella ya está situada. Eso también está muy bien (Girona, mujer, 55 años).*<br>*Había una doctora sustituta (…) en realidad todo ese historial no le interesaba a ella. Yo venía por… yo creo que se lo tuve que explicar. (Girona, mujer, 55 años)*<br>*Cada día que voy hay una persona (médico de cabecera) distinta, porque está de baja (…) Es como un lío. Tengo que volver a contar todo cada vez. (…) (La historia clínica) lo pueden leer, pero no sé, si se lo tienen que leer todo. No sé, ya voy más rápido contándoselo. (Barcelona, mujer, 26 años)*

B. Factors influencing (dis)continuity of clinical management and information across care levels

Category	Quotation
**Factors related to the health system**	
Distribution of roles between physicians of both care levels	*Los medicamentos no los cambia nadie nada más que la nefróloga o la de medicina interna (…) El médico de cabecera es él que tiene que hacer las recetas de crónico, él no ajusta el medicamento. El médico de cabecera solamente lo coordina dijéramos. (Baix Empordà, mujer, 43 años)*<br>*El médico de cabecera está pendiente de las enfermedades crónicas que uno tiene, ¿no? Y ya después de ver cómo vamos, si tenemos alguna recaída. (…) Y una vez que nos remiten al especialista, ya ahí ese tema ya lo tienen ¿cómo decir? evacuado; ya lo tienen a parte. Y tratan otros temas que vengas con problemas de salud. (Girona, hombre, 42 años)*

	Cada uno receta lo que tiene que recetar, desde su especialidad, y el médico de cabecera es el que tiene la versión de todo y bueno, si tiene que hacer alguna recomendación la hace. (Barcelona, hombre, 45 años)<br>*El cardiólogo es el que nos lleva a nosotros cada x tiempo, tenemos la visita ya concertada para llevarla. Y el de cabecera solamente lo que le va (a mi marido) saliendo a diario. Si tiene una infección, si tiene una gripe o si tiene un constipado (…) El de cabecera tiene que llevarla continúa. (Girona, mujer, 82 años)*<br>*La percepción que tengo es esta, que (los médicos de cabecera) quieren evitar enviarte al especialista (…) ahora es como si (el médico de cabecera) quisiera solucionar los problemas sin derivarte al especialista. Entonces, creo que es un campo que se le queda grande, al médico de cabecera, porque saber de todo es como no saber de nada. (Girona, hombre, 47 años)*<br>*Yo creo que estaría mejor que puedas ir directamente al especialista porque es que ahorra mucho tiempo (…) si tú ya ves claro que tienes que ir al especialista, que es una tontería esperar a que el médico te diga "a sí, pues sí, tienes que ir". (Baix Empordà, mujer, 43 años)*
**Factors related to the health services organizations**	
Insufficient resources	*Este de urgencias, que yo pienso que sí que tienen mucho trabajo que quizás van cortos de personal. (…) No sé si ellos abren la ficha de cada persona que va. Dudo que tengan tiempo, porque están bastante abarrotados de gente. (Baix Empordà, mujer, 42 años)*<br>*He visto que bueno, que sí, que el médico de cabecera y los especialistas pues, no he detectado que no haya una coordinación aceptable, ¿no? Para mí sí, ha funcionado, sí, me he sentido atendida (…) Y es que los recortes, lo que van a llevar es que, esta coordinación se rompa. (Barcelona, mujer, 52 años)*<br>*Los médicos especialistas, como puede ser el caso del oftalmólogo, hay pocos para la cantidad de gente que estamos aquí. Lo normal es que si pides hora para el médico de la vista pues te ve para seis meses, que esto es una locura. (Barcelona, hombre, 54 años)*<br>*Si ahora (la sanidad) no funciona bien es porque hay recortes y por que los sociosanitarios y los médicos van desbordados (…) entonces aquí si podemos sufrir todos, tanto los profesionales de la sanidad como los pacientes. (Barcelona, mujer, 52 años)*
Care coordination mechanisms	*Duplicarme pruebas, no, todo lo contrario como todo queda en el ordenador: "oye no te saco sangre porque te han sacado hace un mes." (Baix Empordà, mujer, 41 años)*<br>*No sé desde cuando está informatizado todo esto, pero tienen mucha información (…) análisis y pruebas (…) Yo creo que la coordinación entre los médicos es el ordenador (Baix Empordà, mujer, 52 años)*<br>*Sé que hay reunión porque bueno cuando yo he ido al traumatólogo, pues me ha dicho: "no en la reunión del viernes pasado o el jueves pasado se comentó tu caso" (…) o la doctora de cabecera me ha dicho "el rehabilitador me comentó que en lugar de visitarlo a él pues que yo te cambie la medicación esta", o sea que realmente sí, que hay coordinación. (Baix Empordà, hombre, 41 años)*

	Sé que hay reunión (…) realmente se conocen entre ellos y hablan los casos y sí (…) menos mal, porque así no es que te mandan a un especialista y el especialista tal no sabe ni quién es la doctora de cabecera o el médico que te ha mandado. (Baix Empordà, hombre, 41 años)
	Ellos también se hablan en diferentes ocasiones. A veces he hablado con la doctora mía y dice: "sí, sí, he hablado con fulano de tal, he hablado con este, con el otro, pensamos que esto es así", se hablan (….) luego los resultados, al momento sabes lo que tienes, no te hacen esperar. (Baix Empordà, hombre, 61 años)
	Ahora con ese tema de la tiroides, la doctora dice 'a mí, me parece que es eso pero lo voy a consultar con la endocrinóloga' e hizo una consulta teniendo en cuenta que yo había tenido esta enfermedad. (…) En la próxima visita me informó de que le había mandado un correo y la otra ya le había contestado, pero en un plazo relativamente, bien, correcto. (Girona, mujer, 55 años)
	Yo cuando voy a cardiología, él abre mi número y sabe toda la medicación que tomo (…) La de cabecera igual, lo sabe todo, aprieta el botón, en la ficha sale todo. Además miran que sea compatible una medicación con otra. (Barcelona, hombre, 70 años)
Co-location of physicians	*Supongo que sí (hay comunicación) porque yo sé que las rehabilitadoras van a los CAPs porque cuando yo estuve mal de la espalda, a la rehabilitadora le dije: "tengo que subir con el informe al del cabecera", y me dijo "no, porque nos comunicamos", o supongo que si ella abre el ordenador también lo ve. (Baix Empordà, mujer, 42 años)*
	Al ser en el mismo ambulatorio los resultados los tienen en el mismo ordenador. Los ordenadores están interconectados dentro del ambulatorio (…) los comentarios que me hace (el otorrino) pues los va escribiendo ella en el ordenador (…) cuando voy al médico de cabecera lo mira en el ordenador y me dice "sí, sí, ya veo lo que me había comentado". (Barcelona, hombre, 48 años)
	La fisio (en el CAP) me dijo: "oye, ¿estás bien?" Y yo "¿cómo lo sabes tú esto? (…) "porque puedo leerlo todo (…) lo podemos leer tu médico de cabecera y yo." (Barcelona, mujer, 26 años)
**Factors related to the physicians**	
Willingness to collaborate	*Si vas al especialista y el médico ya te ha hecho una clase de pruebas como por ejemplo hacerte radiografía o algo, ya te dice que te la lleves (…) para que la diera al especialista (…) para no tenerla que volver a hacer. (Girona, mujer, 64 años)*
	El médico de cabecera en principio (…) querría que me hiciese pruebas (de análisis de sangre) un par de veces al año. Pero como ya me las van haciendo en el Hospital del Mar, pues simplemente con llevarle los resultados ya tiene suficiente, por no hacer las mismas pruebas dos veces, porque van a mirar exactamente lo mismo. (Barcelona, hombre, 48 años)
	(El neurólogo) me dice o bajar la dosis de ese medicamento o cambiarlo por otro, siempre pues intentando hacer caso del médico de cabecera (…) El neurólogo pues viendo el medicamento que me había aconsejado la doctora pues dijo: "vale, pues te quitamos este que ya no te hace efecto." (Barcelona, hombre, 48 años)

Commitment to the patient care	*He estado 30 años en Barcelona y más o menos, sí (hay comunicación), pero aquí todo el mundo se conoce más. Los médicos se consultan mucho, porque es un interés que el paciente esté bien, o sea aquí las prioridades es esta, que la persona esté bien. (Baix Empordà, mujer, 33 años)*<br>*El especialista es el que tendría la obligación de tratar a fondo (…) tiene que hacer el especialista del área de la cabeza, y de esa forma, pues, poderse entre los médicos, unirse y poder investigar, ¿no? (…) No miro interés de solucionar el problema, si no de ¿cómo decir? de salir del paso. ¿Hay x problema? Pues pongamos esto y ya está. (Girona, hombre, 42 años)*<br>*Soy un número más para ella (médico de cabecera) y ya está. Es lo que siento (…) porque cuando yo me fui por lo de mi pierna, no yo sentí ningún... que ella tuviera un poquito de interés como para buscar a ver si hay un turno antes ¿sabes? Directamente me lo dijo: "no", así rotundamente (…) porque yo le he preguntado si me podían dar para diciembre, porque yo quería saber qué era lo que tenía en mi pierna ¿sabes? (Barcelona, mujer, 37 años)*
Primary care physician's technical competence	*Él de cabecera, es el que tiene el conocimiento. Primero es el de cabecera, él sabe a quién ha de llamar o pedir hora de visita (…) muy bien. (Baix Empordà, hombre, 81 años)*<br>*El medico de cabecera es el que hace la valoración y considera si realmente es necesario de que te visite (el especialista) o no, muy bien. (Baix Empordà, mujer, 46 años)*<br>*Si yo tengo un problema x, yo no sé qué problema tengo. Entonces no sé si es cuestión de ir al cardiólogo o al traumatólogo, por decir algo. No sé dónde debo ir (…) Alguien tiene que dirigir esto, y es el médico de cabecera. (Barcelona, hombre, 55 años)*
